# Supplementary figures and images for: Integrated Analysis of Metabolomics, Flavoromics, and Transcriptomics for Evaluating New Varieties of Amomum villosum Lour
Source: Plants (Basel). 2024 Aug 26;13(17):2382. doi: 10.3390/plants13172382 (PMC11397242; doi:10.3390/plants13172382)

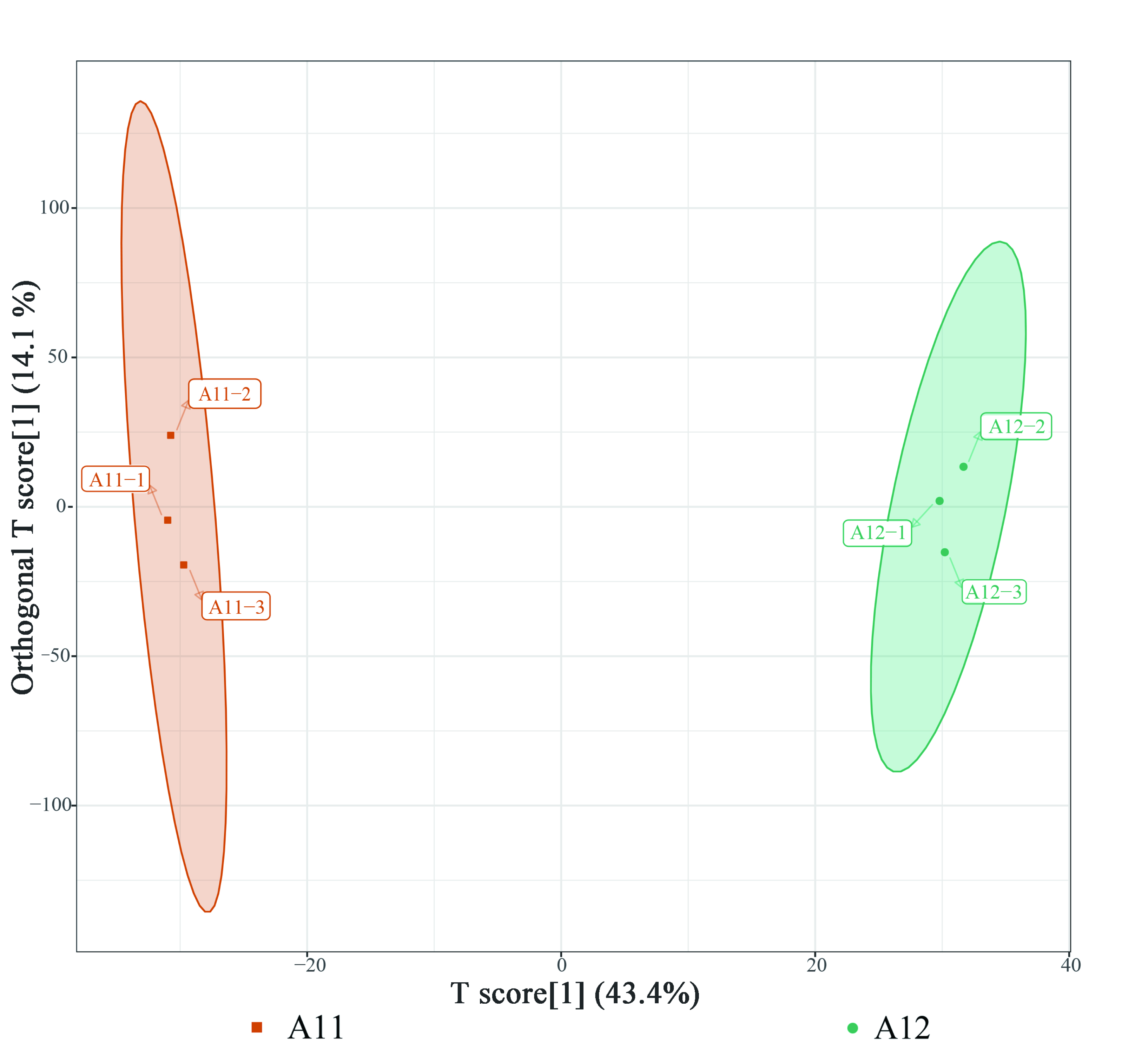

Supplement: Supplementary file 1 [file plants-13-02382-s001.zip › Supplementary Figure S1.tif]

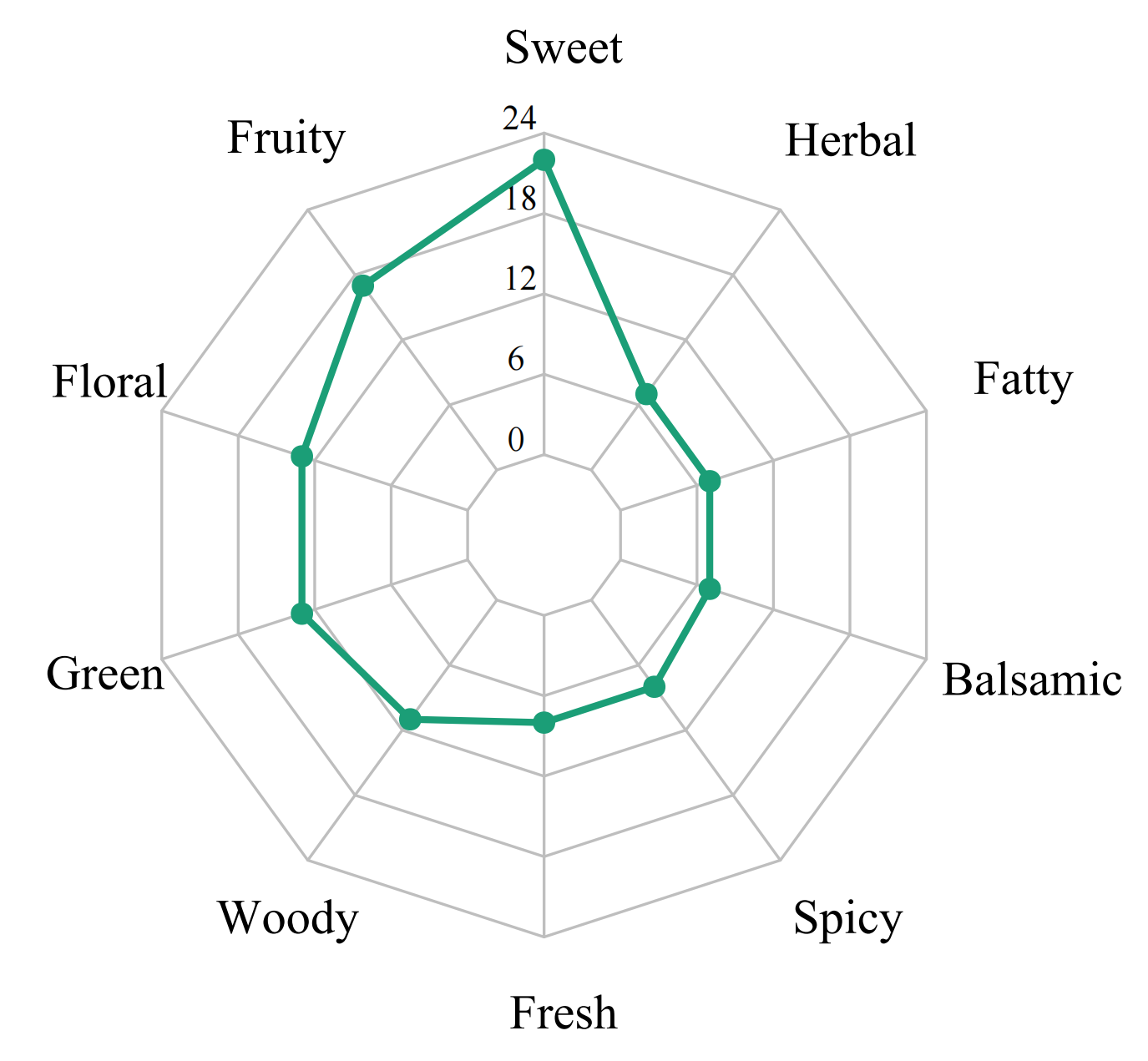

Supplement: Supplementary file 1 [file plants-13-02382-s001.zip › Supplementary Figure S2.tif]
